# Development of Auditory-Vocal Perceptual Skills in Songbirds

**DOI:** 10.1371/journal.pone.0052365

**Published:** 2012-12-20

**Authors:** Vanessa C. Miller-Sims, Sarah W. Bottjer

**Affiliations:** Section of Neurobiology, University of Southern California, Los Angeles, California, United States of America; Baycrest Hospital, Canada

## Abstract

Songbirds are one of the few groups of animals that learn the sounds used for vocal communication during development. Like humans, songbirds memorize vocal sounds based on auditory experience with vocalizations of adult “tutors”, and then use auditory feedback of self-produced vocalizations to gradually match their motor output to the memory of tutor sounds. In humans, investigations of early vocal learning have focused mainly on perceptual skills of infants, whereas studies of songbirds have focused on measures of vocal production. In order to fully exploit songbirds as a model for human speech, understand the neural basis of learned vocal behavior, and investigate links between vocal perception and production, studies of songbirds must examine both behavioral measures of perception and neural measures of discrimination during development. Here we used behavioral and electrophysiological assays of the ability of songbirds to distinguish vocal calls of varying frequencies at different stages of vocal learning. The results show that neural tuning in auditory cortex mirrors behavioral improvements in the ability to make perceptual distinctions of vocal calls as birds are engaged in vocal learning. Thus, separate measures of neural discrimination and behavioral perception yielded highly similar trends during the course of vocal development. The timing of this improvement in the ability to distinguish vocal sounds correlates with our previous work showing substantial refinement of axonal connectivity in cortico-basal ganglia pathways necessary for vocal learning.

## Introduction

Songbirds, like humans, learn the vocal sounds used for communication during a sensitive period early in life. Processes of vocal learning between humans and songbirds are strikingly similar: juveniles memorize sounds from vocal “tutors” during an auditory memorization phase, and subsequently translate that memory into a motor program by learning to match feedback of their incipient vocalizations (babbling) to the auditory memory of tutor sounds during a phase of sensorimotor integration [1], [2]. Vocal communication involves acquiring the ability to both produce and perceive learned sounds. Studies of human infant vocal learning have shown that developmental improvements in perception of speech sounds precede those in production, and predict later success in speech and language acquisition [3], [4], [5], [6]. In contrast, studies of juvenile songbirds have focused on the influence of auditory experience on vocal production [7], [8], [9], and the development of vocal perception has been little studied.

**Figure 1:**
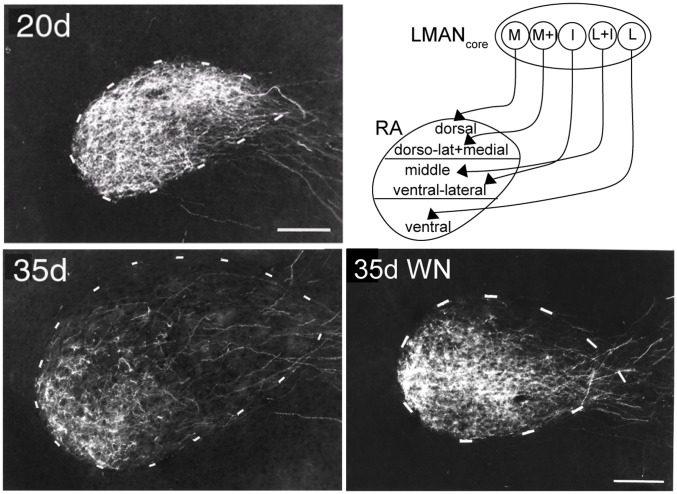
Remodeling of the axonal projection from a cortico-basal ganglia circuit to vocal motor cortex in zebra finches. The core region of LMAN (lateral magnocellular nucleus of the anterior nidopallium) provides the cortical outflow of a basal ganglia circuit necessary for vocal learning to vocal motor cortex (RA, robust nucleus of the arcopallium). Previous work from our lab has shown that the axons of LMAN_core_ form a precise topographic map in RA of adult birds (shown schematically in the upper right panel), which may represent a myotopic map of vocal muscles and/or acoustic features or vocal sounds. However, the axonal projection from LMAN_core_ to RA has little or no topographic organization in 20-day birds at the onset of vocal learning (20 d, upper left panel): axons from small subregions of LMAN_core_ spread throughout most of RA at this age [24], [69]. However, LMAN_core_ axons of birds that receive normal auditory experience undergo substantial re-modeling to form a normal topographic map by 35 days of age (35 d, lower left panel) [24]. In contrast, this axonal remodeling is prevented in juvenile birds that are raised in constant white noise [24]. This remodeling is due to both pruning and re-positioning of individual axons, including both genetic and experience-dependent changes in neural connectivity during the first two weeks of vocal learning [25]. Scale bars = 200 um.

A fundamental question for all models of vocal learning pertains to the relationship between processes of vocal learning and the development of perceptual abilities [10]. Human infants begin life with the ability to distinguish phonetic contrasts of all languages but perceptual discrimination of non-native phonemes declines by 6–12 months of age [11], [12], [13]. In addition, infants’ ability to discriminate between native language sounds improves [14], [15]. This enhanced perceptual ability for sounds experienced in the auditory-vocal environment occurs as infants are forming a memory of those sounds and has been suggested to result from refinement of neural connections, resulting in an irreversible “neural commitment” [16], [17]. Refinement of neural connectivity is enhanced at all levels of auditory circuitry from brainstem to cortex during specific windows of development across taxa [18], [19], [20], [21], [22], [23]. No studies to date have investigated developmentally-regulated plasticity in higher-order regions of songbird auditory cortex and their connections to sensorimotor vocal-control regions during vocal learning. However, previous work from our lab has demonstrated dramatic remodeling of axonal connections in sensorimotor brain regions that underlie vocal learning during the developmental time period when juvenile songbirds are learning tutor sounds and gradually producing correct imitations of them (Fig. 1) [24], [25]. This pruning and rearrangement of neural connections is dependent on normal auditory experience, suggesting that this refinement represents specific vocal patterns stored in memory – a form of neural commitment.

Assays of vocal perception in animal models are essential for investigating how learning and experience-dependent changes in neural processing contribute to the emergence of perceptual skills. No studies to date in songbirds have investigated whether the ability to discriminate between conspecific vocal sounds changes throughout the course of vocal development (but see [26]). In infants, behavioral assays of perception are common, and neural measures have been shown to provide sensitive measures of speech discrimination that correspond to behavioral measures [14]. For example, event-related potentials in response to native versus non-native phonemic contrasts demonstrate an increase in responsivity to native-language contrasts between 7 and 11 months, consistent with behavioral data [27]. In contrast, studies in animal models have tended to focus primarily on dependent measures in the CNS as a function of auditory experience, with far less attention paid to behavioral measures of perceptual development [22].

In order to exploit the strength of songbirds as a model system for human speech and provide direct tests of relationships between learning, perception, and their neural substrates, we developed both behavioral and neural assays of discrimination of vocal sounds that can be applied across development. These assays are modeled after procedures used in studies of speech perception in human infants; the same tasks were used at all ages, thus allowing changes in the ability to distinguish vocal sounds to be examined during the period when birds are engaged in the processes of song learning from juvenile to adult stages. The results demonstrate that, as in humans, both behavioral and neural measures of discrimination of “native-language” sounds improve during the course of vocal development [3], [14], [15]. These data constitute an essential step in advancing our ability to dissect relations between perception and production, and in relating perceptual behavior and neural measures to vocal learning across development.

## Results

Zebra finches use “long calls” to communicate between partners and between parents and offspring, especially over longer distances when visual contact is eliminated [28], [29], [30], [31], [32]. Long calls are complex sounds that have a fundamental frequency (FF) with several harmonics (Fig. S1), and are used for individual recognition [32], [33], [34], [35]. Females produce long calls with lower FF than males and each individual produces a call with a characteristic FF [30], [31], [36], [37]. Experimental shifts in the FF of female calls influence behavioral responsivity of males [38], consistent with the idea that the pitch of calls is an important acoustic variable [33], [39]. We tested the ability of male birds at different stages of vocal learning to make distinctions between long calls of different FFs: 20-day birds that have just left the nest and are beginning to engage in vocal learning; 35-day birds that have memorized tutor vocal sounds and are beginning the phase of vocal babbling; and young adults (100 days of age) that are producing stereotyped imitations of tutor sound. The same tests of discriminative ability were applied at each age.

A habituation-dishabituation paradigm was used to conduct both behavioral and neural tests of how well birds could discriminate synthetic long calls that varied in fundamental frequency. A single call stimulus was presented repeatedly until the response (either behavioral or neural) habituated, followed by randomized presentations of 6–8 novel test stimuli interleaved with the habituated stimulus. Recovery of response to a test stimulus (dishabituation) indicates that that stimulus can be discriminated from the habituated stimulus, whereas a failure of response recovery indicates an inability to distinguish that test stimulus from the habituated stimulus [11], [40], [41]. All stimuli presented were synthetic long calls consisting of harmonic stacks that spanned the range of natural male and female calls recorded from our breeding population (Fig. S1). The habituating stimulus had a FF of either 480, 650 or 800 Hz and test stimuli consisted of pitch-shifted calls in which the fundamental frequency was increased or decreased in steps of 20–50 Hz (see Methods).

The behavioral assay relied on the fact that zebra finches respond to long calls by producing a long call of their own (call-back behavior) [42], [43], [44]. The neural assay measured the multi-unit electrophysiological responses of awake birds within NCM (caudo-medial nidopallium), a region of higher-level auditory cortex that is highly responsive to vocal sounds [45]. Previous studies have shown that NCM neurons respond well to complex stimuli such as conspecific songs and calls, and may encode a neural representation of the tutor song learned during the sensitive period; in addition, NCM includes neurons that habituate to repeated presentations of complex sounds [46], [47], [48], [49], [50] (Fig. S2). Recent work also shows that neural activity in NCM matches behavioral performance when songbirds are trained to discriminate among vocalizations [51]. Thus, NCM represented a logical region to examine as a locus for possible neural correlates of perceptual ability.

### Both Behavioral and Neural Assays of Discriminative Ability Show Developmental Improvements

**Figure 2:**
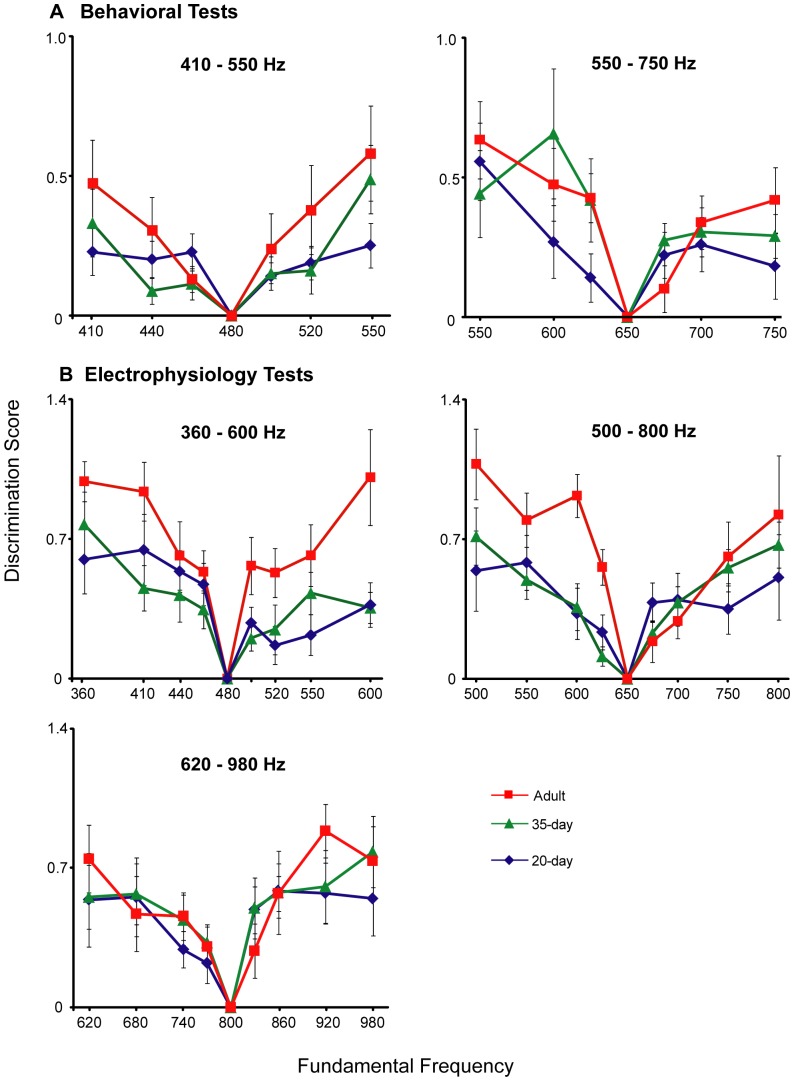
Discrimination gradients depicting ability to distinguish vocal calls for birds of different ages. (A) Behavioral discrimination scores (mean ± SEM) are plotted for three age groups of birds that were habituated to a call stimulus with a FF of 480 Hz and then tested with novel calls ranging in FF from 410 to 550 Hz (left panel); three separate age groups were habituated to a call stimulus of 650 Hz and tested with novel calls ranging from 550 to 750 Hz (right panel). (B) Neural discrimination scores (mean ± SEM) for three separate experiments in which different age groups were habituated to call stimuli with a FF of either 480, 650, or 800 Hz and then presented with novel test calls that varied in FF. Discrimination score: 0.0 = no different from habituated stimulus, 1.0 = completely discriminated; see Methods.

Both call-back behavior and neural activity in NCM showed habituation to repeated presentations of a synthetic call stimulus followed by dishabituation to presentations of novel call stimuli [45], [50]. We computed a discrimination score to quantify how well birds could distinguish novel test stimuli from the habituated stimulus for both the behavioral and neural tests; this score varied from 0.0 for no discrimination to 1.0 for perfect discrimination (see Methods). Fig. 2A shows mean discrimination scores for two separate behavioral tests of perception in which the FF of the habituating stimulus was either 480 Hz (left panel) or 650 Hz (right panel). The pattern of dishabituation showed that birds of all ages were able to distinguish novel test calls from the habituated call, and that discriminative ability was greater for test stimuli farther away from the habituated stimulus. Adults tended to show relatively steep discrimination gradients: test stimuli farthest from the habituated stimulus were strongly dishabituated. Juvenile birds showed flatter discrimination gradients; this was particularly true for 20-day birds, which tended to show low levels of dishabituation for stimuli far from the habituated stimulus (e.g., to 550 Hz in the first test).

Results from three separate electrophysiological tests were similar to those from the behavioral tests: response strength in NCM was larger the farther the test stimulus was from the habituated stimulus, and adult birds tended to show steeper discrimination gradients than juveniles (Fig. 2B; FF of the habituating stimulus was either 480, 650, or 800 Hz). Adult birds that were habituated to either the 480 Hz or the 650 Hz stimulus had scores close to 1.0 at the farthest FF (120–150 Hz from the habituating stimulus), indicating strong neural discrimination of calls further removed in frequency, whereas 20-day birds had scores close to 0.5 at these same frequencies. Adult birds habituated to the 800 Hz stimulus had somewhat lower scores (∼0.8) at the furthest FF (180 Hz from the habituating stimulus) suggesting that discriminative ability of NCM neurons is not uniform across all frequency ranges (see below).

**Figure 3:**
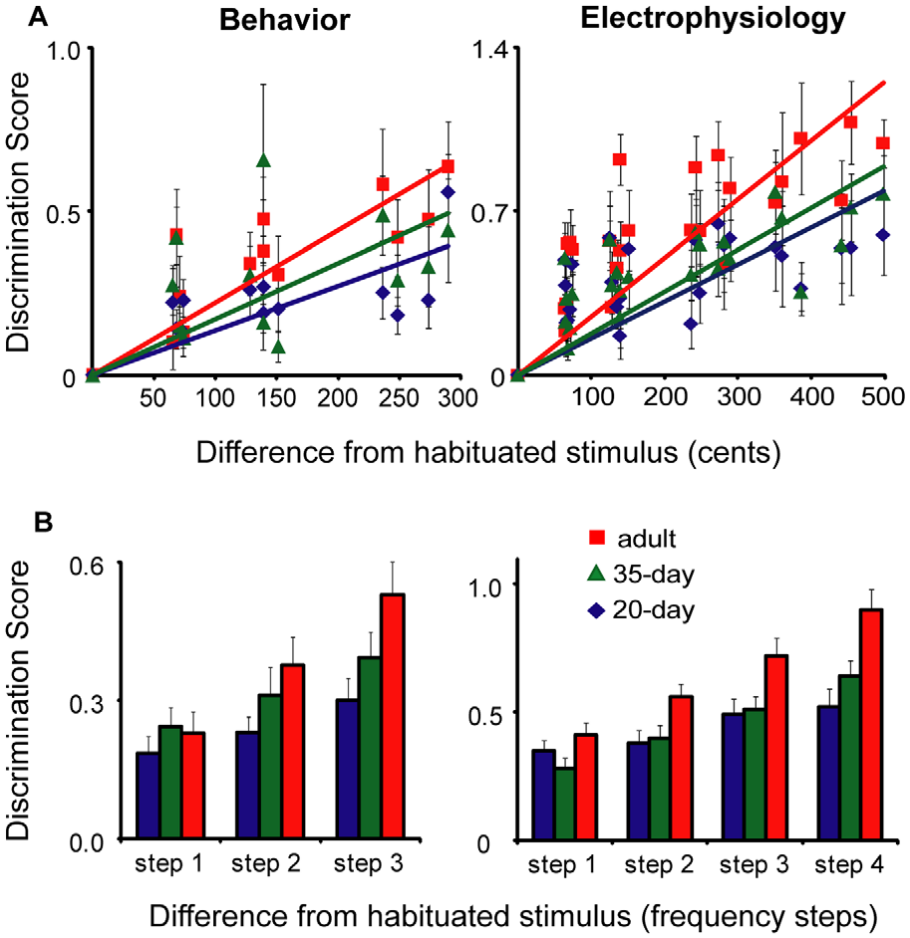
Steepness of discrimination gradients as a function of age. (A) Data from Fig. 2 plotted as psychometric functions showing discrimination score versus cents difference from habituated stimulus (both higher and lower). Trendlines for each graph have y-intercept set to 0 for ease of comparison (see Methods). (B) Discrimination scores for each age group at increasing steps of frequency away from the habituated stimulus (see Results). For both A and B, behavioral data are plotted in the left panels, and neural data from NCM are plotted in the right panels. All values are mean ± SEM.

We quantified the steepness of the discrimination gradients shown in Figure 2 by plotting the discrimination scores as a function of the difference in FF between each test stimulus and the habituated stimulus, expressed in cents (Fig. 3A). A regression analysis for all data points from both behavioral tests demonstrated a significant correlation between discrimination scores and distance from the habituated stimulus at all three ages (20-day: F_1,117_ = 20.6, *p*<0.0001, R = 0.387; 35-day: F_1,104_ = 16.7, *p*<0.0001, R = 0.372; adult: F_1,118_ = 32.9, *p*<0.0001, R = 0.467). Likewise, a regression analysis based on NCM activity in all three neural experiments revealed that the neural discrimination scores showed a significant correlation with distance from the habituated stimulus at all three ages (20-day: F_1,214_ = 22.7, *p*<0.0001, R = 0.310, 35-day: F_1,222_ = 59.2, *p*<0.0001, R = 0.457, adult: F_1,223_ = 76.3, *p*<0.001, R = 0.505).

The slopes of the linear regression lines shown in Figure 3A for the behavioral experiments were 0.48, 0.59, and 0.91 for 20-day birds, 35-day birds, and adults, respectively (see Methods). The slope of the regression based on NCM activity in the neural experiments was also higher in adult birds (0.86) compared to juveniles (0.43 and 0.61 in 20-day and 35-day birds, respectively). To quantify this difference in ability to discriminate how different one stimulus is from another between age groups we examined mean discrimination scores at increasing frequency steps from the habituated stimulus. For each experiment, the first step was defined as the two test stimuli (higher and lower frequencies) closest to the habituated stimulus; the second step included the next closest frequencies, etc.

In the behavioral tests (Fig. 3B, left) adult birds showed larger increases in discrimination scores for test stimuli further from the habituated stimulus compared to juvenile birds. Thus, adults showed better perceptual discrimination once a test stimulus was sufficiently different from the habituated stimulus. Within-subject Friedman tests showed that 20-day birds showed modest, but non-significant improvement at larger frequency steps (χ^2^ = 4.0, *p* = 0.13). In contrast, adult birds showed progressively higher scores across steps (χ^2^ = 14.8, *p* = 0.001). Performance in 35-day birds was intermediate between 20-day birds and adults at steps 2 and 3; although scores at this age tended to improve for larger frequency steps, the effect was not significant (χ^2^ = 4.1, *p* = 0.13). A Kruskal-Wallis test for the main effect of age showed a significant effect on behavioral discrimination scores only at the third step (χ^2^ = 6.0, *p* = 0.05), reflecting the enhanced ability of adult birds to distinguish frequencies further removed from that of the habituating call. Individual Mann-Whitney comparisons showed a significant difference between 20-day birds and adults at the third step (*p* = 0.02), but 35-day birds were not significantly different from either 20-day birds (*p* = 0.17) or adults (*p* = 0.27). Discrimination scores were significantly different from zero at all steps and all ages (one-sample Wilcoxon signed-rank tests, *p*<0.001 in all cases).

The results of the electrophysiological tests showed similar trends to the behavioral experiments (Fig. 3B, right). Neural discrimination scores of 20-day birds showed mild but nonsignificant improvement from the first to the fourth step (Friedman test, χ^2^ = 4.9, *p* = 0.18). In contrast, the ability of NCM neurons in adult birds to discriminate between calls improved substantially: mean discrimination scores more than doubled from the first to the fourth step (χ^2^ = 26.1, *p*<0.0001). Neural discrimination scores of 35-day birds also showed a significant increase in performance over the four steps (χ^2^ = 50.5, *p*<0.0001). A Kruskal-Wallis test for the main effect of age was significant for each of the last three steps (step 1, χ^2^ = 3.7, *p* = 0.16; step 2, χ^2^ = 7.7, *p = *0.02; step 3, χ^2^ = 8.6, *p* = 0.01; step 4, χ^2^ = 13.8, *p* = 0.001). Individual Mann-Whitney comparisons showed no significant difference between 20- and 35-day birds at any step (*p*>0.18 for steps 1–3; *p = *0.07 for step 4). Neural discrimination scores in adults were significantly higher than those of either 20-day or 35-day birds at each of the last 3 steps (*p*<0.02 in all cases). At all steps and all ages the discrimination scores were significantly different from zero (*p*<0.001 in all cases).

In summary, neural and behavioral tests showed remarkably similar results: younger birds showed flatter regression lines than adults with lower correlation coefficients, indicating a diminished ability to discriminate calls of different frequencies at the onset of the sensitive period for vocal learning. Juvenile birds showed relatively low levels of dishabituation to frequencies that were far from the habituated stimulus, and hence showed less steep discrimination gradients compared to adults: adults were able to respond to a call 120–150 Hz from the habituated stimulus as if it were completely novel whereas the response of 20-day birds showed less than 50% dishabituation at this distance. However, the discrimination score was significantly different from zero at all points for all ages, indicating that juvenile birds have some ability to discriminate calls that vary in FF, but are less able to assess how different one call is from another than are adults. Thus, the behavioral ability to discriminate between long calls of different FFs improves between the onset of vocal learning at 20 days and adulthood, indicating an improvement in the ability to perceptually distinguish vocal calls. The similar age course in the ability of NCM neurons in awake birds to discriminate between calls of different fundamental frequency suggests that NCM may be an important part of the neural substrate for this developmental refinement in perception.

### Neural Discrimination in Adult Birds is Better for Calls with Lower Frequencies

**Figure 4:**
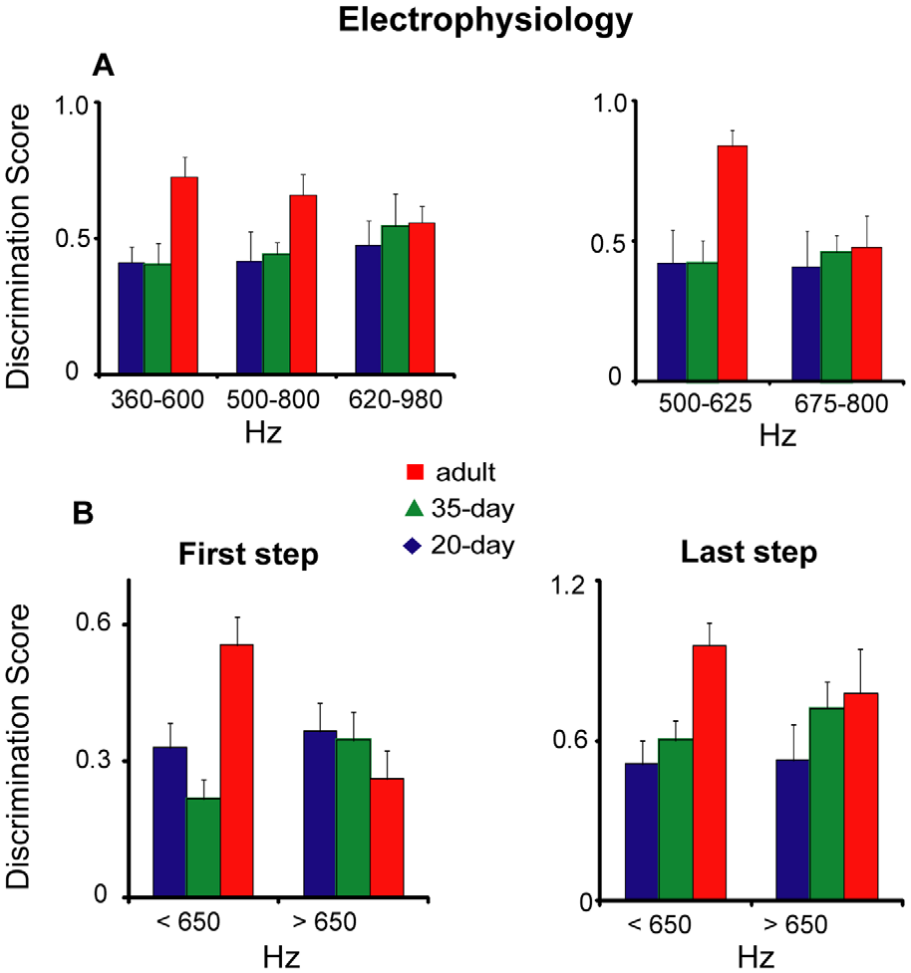
Neural discrimination gradients as a function of age and frequency range. (A) Discrimination scores averaged across all frequencies within each frequency range (left panel) and separately for test stimuli below versus above the habituating stimulus for the experiment in which birds were habituated to a call of 650 Hz and tested with calls from 500–800 Hz. (B) Discrimination scores at the first and last frequency steps within the high (675–800 Hz) versus low (500–625 Hz) frequency ranges. All scores plotted as mean ± SEM.

To determine whether discriminative ability of NCM neurons varied within different frequency ranges we averaged discrimination scores for each bird across the test frequencies within each of the three electrophysiological experiments depicted in Fig. 2B. Fig. 4A (top left) shows these data for each age group in each frequency range; mean neural discrimination scores did not vary across the three frequency ranges tested for 20- and 35-day birds. In contrast, adults showed enhanced discriminative ability in the lower frequency ranges (360–600 and 500–800 Hz). Age exerted a significant effect on discrimination scores only for the 360–600 Hz frequency range (Kruskal Wallis, χ^2^ = 8.4, *p* = 0.02), reflecting the fact that adult birds had significantly higher scores than both groups of juveniles (20-day versus adult: *p* = 0.01; 35-day versus adult: *p* = 0.02). However, the absence of a significant effect of age in the middle frequency range (500–800 Hz) is complicated by the fact that the discrimination gradient for adult birds was asymmetrical in this range (Fig. 2B): discriminative performance was steeper at lower frequencies (<650 Hz) than at higher frequencies (>650 Hz). We therefore calculated the average discrimination scores in this range for 500–625 Hz versus 675–800 Hz (Fig. 4A, top right), and found a significant effect of age in the lower frequency range (χ^2^ = 9.9, *p* = 0.007) but not in the higher range (χ^2^ = 0.1, *p* = 0.95), again reflecting higher discrimination scores in adults birds compared to juveniles (20-day versus adult: *p* = 0.02; 35-day versus adult: *p* = 0.003). Within-subject tests (Wilcoxon signed-ranks) revealed no difference between these two frequency ranges in 20-day (Z = −0.56, *p* = 0.58) or 35-day (Z = −0.18, *p* = 0.86) birds whereas adult birds showed substantially higher discrimination scores in the lower range than in the higher range (Z = −2.4, *p = *0.02).

Figure 3B shows that discriminative performance at the first frequency step (closest to the habituating stimulus) was the same across ages when all frequencies were combined across tests (for behavioral as well as neural measures). Similarly, Figure 4B (left) shows no age difference in neural discrimination between calls at the first step in the high frequency range (675–600 Hz; χ^2^ = 2.4, *p = *0.31). In contrast, age exerted a highly significant effect at the first step for frequencies between 500 and 625 Hz (χ^2^ = 14.2, *p* = 0.001). Similar results were found at the last frequency step; there was a significant effect of age on neural discrimination in the low-frequency range (χ^2^ = 15.4, *p*<0.0001) but not the high-frequency range (χ^2^ = 2.2, *p* = 0.33; Fig. 4B right). Thus, regardless of the difficulty of discrimination, NCM neurons of adults are better at discriminating between calls in the lower frequency range than are those of juveniles (as seen in Fig. 2B, 360–600 Hz). At the onset of song learning, the ability of NCM neurons in 20-day birds to discriminate between calls of different frequencies is similar across all frequency ranges. By adulthood NCM neurons develop an enhanced ability to discriminate between calls below 650 Hz (Fig. 2), which includes the range of FF for female calls (Fig. S1). This pattern suggests that male birds’ perception of vocal calls of different FFs may become optimized to discriminate between female calls during the course of vocal learning.

**Figure 5:**
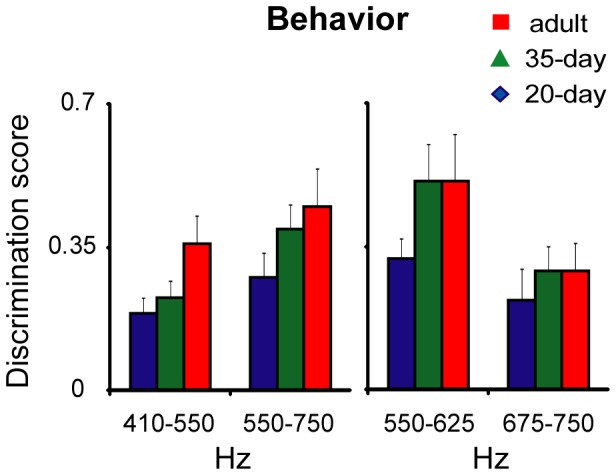
Behavioral (call-back) discrimination scores vary as a function of age and frequency range. Left, discrimination scores averaged across all frequencies within reach behavioral experiment for each age (mean + SEM). Right, discrimination scores from the 550–750 Hz test are re-plotted for low-frequency (550–625 Hz) versus high-frequency (675–750 Hz).

The results of the behavioral tests were largely similar to those for the electrophysiology tests. The left panel of Figure 5 shows better perceptual discrimination by adult birds than by juveniles in the 410–550 Hz frequency range, but less of an age difference in the range of 550–750 Hz. The effect of age on frequency discrimination within the 410–550 Hz range was marginally significant (*p* = 0.06), but age had no significant effect within the 550–750 Hz range (*p* = 0.13). The right panel of Figure 5 presents average discrimination scores from the 550–750 Hz experiment separately for 500–625 Hz versus 675–750 Hz. These data show a similar trend to that seen for neural discrimination in the high-frequency (675–800 Hz) range: adults and 35-day birds showed better discrimination in the lower frequency range than in the higher frequency range. However, there was no significant effect of age within either frequency range (low *p* = 0.17; high *p* = 0.58). In summary, adult birds showed better perceptual discrimination in the lower frequency range compared to the higher frequency range whereas 20-day birds showed similar levels of discrimination across frequency ranges. However, in contrast to the neural tests, 35-day birds in the behavioral experiment showed better discrimination of calls in the 550–625 Hz range compared to the 675–750 Hz range, similar to the pattern for adults.

### Evaluating Developmental Differences in Discriminative Ability

A general concern is whether the responses of juvenile and adult birds measure discriminative ability in an equivalent fashion (cf. [52]). For example, it is unlikely that 20- and 35-day birds produce a call-back response identical in nature to that of adults, since zebra finches become sexually mature ∼90 days. We assessed this possibility by examining the baseline rate of call-back responsivity at different ages. Figure S3 shows the number of call-backs given in response to adult male versus female calls as well as to each synthetic stimulus (these birds were tested specifically for this purpose and were not included in the habituation-dishabituation tests). These control data show that 20-day birds produced the highest number of call-backs (average of 37.5 across all stimuli), whereas 35-day birds produced the lowest number (average of 6.1) and adult birds were intermediate (average of 12.7) (Kruskal-Wallis, χ2 = 8.5, p = 0.02). In addition, only adults called back more to female calls than to male calls as demonstrated previously by Vicario and colleagues [28], [43].

This pattern suggests that juvenile birds (especially 20-day birds) do not respond to playback of synthetic calls in the same way as adults. Our strategy to control for a possible influence of age differences in responsivity in the behavioral tests was two-fold. Firstly, we set a habituation criterion (failure to respond to 2 of 3 stimuli; see Methods) instead of playing the same number of habituation stimuli for each bird. Thus, the same criterion of habituation was applied across all age groups, so that all birds reached a similar level of habituation prior to testing (Fig. S4). Second, we calculated a discrimination score in which the level of dishabituation during testing was normalized to the number of calls initially produced during the habituation phase, and thus each bird served as its own control such that individual differences in rate of call-back had little or no influence on the normalized discrimination score.

We also examined the number of trials to criterion and the rate of call-back response during the habituation phase in experimental birds (i.e., those that were tested for dishabituation to novel calls immediately after reaching the habituation criterion; Fig. S4). These data confirmed that the rate of call-back is higher in 20-day birds compared to 35-day birds and adults. As expected, 20-day birds required a larger number of stimulus presentations to reach criterion for the 480-Hz habituation stimulus than 35-day birds or adults, although the main effect of age was not significant (p = 0.08). Surprisingly, adult birds required a larger number of 650-Hz call presentations to reach the habituation criterion compared to 20- and 35-day birds, but the effect of age was also non-significant (p = 0.13). The right panels of Figure S4 show the average number of calls produced in each age group on the first trial and then at each quartile point throughout the habituation training phase. The initial number of call-back responses given to the habituation stimulus in both the 480-Hz and 650-Hz tests was similar between 20-day birds and adults, whereas 35-day birds produced fewer calls at the onset of habituation training. However, 20-day birds maintained a higher level of call-back responding throughout most of the habituation phase. In contrast, the call-back rate of adult birds quickly dropped to match the 35-day rate. By definition, all age groups converged by the end of habituation training as each bird met the criterion. The main effect of age was significant only at the first and third quartiles (p = 0.04 and p = 0.02 for 25% and 75%, respectively) in the 480-Hz test. In the 650-Hz test the effect of age was significant for the initial call response (*p* = 0.01) and the first (p = 0.03) and second (p = 0.05) quartiles. Planned comparisons showed that the call-back rate in 20-day birds was higher than that of both 35-day birds and adults in all cases in which the main effect of age was significant, whereas there was no difference between 35-day birds and adults.

This pattern argues against the possibility that age differences in call-back behavior significantly influenced the test results. One possibility is that decreased attention to long calls in juvenile birds could reduce dishabituation scores and give a spurious indication of lower discriminative ability. However, the enhanced call-back response of 20-day birds suggests that the lower discriminative performance in these birds was not due to attentional factors; indeed the increased tendency of 20-day birds to call back (Fig. S3, S4) suggests that younger birds should show higher levels of dishabituation – an effect opposite to the relatively low levels of discrimination shown in Fig. 2. Alternatively, it could be the case that basic age differences in the proficiency to show behavioral dishabituation might bias even normalized measures of discrimination. The results shown in Figures 2 and 3 are inconsistent with this idea, since birds of all ages tended to show similar discriminative performance at frequency steps closest to the habituating stimulus, as described above (Figs. 2, 3). Lastly, it should be noted that the overall patterns in Figure S4 do not indicate any correlations between the pattern of habituation and the results of dishabituation testing. For example, adult and 35-day birds showed similar patterns during the habituation phase, despite the fact that adult birds showed sharper discrimination gradients (Figs. 2, 3).

Another factor that could potentially bias habituation-dishabituation responses and thus skew measures of discrimination is an inherent tendency to respond differentially to playback of calls with different FFs. Figure S3 shows the baseline rate of call-back responses to the different frequency calls used in our tests at each age. These data show that each bird, at each age, responded with a comparable number of calls across different synthetic stimuli (i.e., no test stimulus evoked a disproportionate response based on innate tendencies). Thus, the dishabituation response to synthetic calls of different FF during testing is not due to birds calling back preferentially to calls of specific frequencies. In addition, it is unlikely that differences between juvenile and adult birds in the ability to discriminate between synthetic calls are due to differences in absolute hearing thresholds, since auditory brainstem responses show that auditory sensitivity is similar in 20-day birds and adults across frequencies [53].

As in the case of the behavioral tests, it is possible that developmental differences in neural responsivity of NCM neurons could potentially bias the results of dishabituation testing. Figure S5 shows that the response of NCM neurons did not vary across age or frequency of the habituating stimulus during the first five stimulus presentations. Although NCM of juvenile birds showed greater response suppression by the end of habituation training, if anything that might provoke a bias in the direction of greater dishabituation unless the general tendency of NCM neurons to respond was somehow diminished. However, the latter idea is difficult to reconcile with the finding that neural discrimination scores at different ages were comparable at the first frequency step (closest to the habituated stimulus) across ages.

Thus, although we cannot rule out age differences in responsivity to playback of vocal calls, the pattern of data presented in Figs. S3, S4, S5 argue against this factor as an important influence. The use of habituation and dishabituation relative to the initial response to a long call has the advantage of using the same test across all ages, and our measure of discrimination, which used a within-subject score in which each bird served as its own control, provides an effective means of assessing whether birds of different ages are able to distinguish vocal sounds of different fundamental frequencies.

## Discussion

Both the behavioral and neural assays used here show that adult zebra finches are substantially better than juveniles in their ability to discriminate vocal calls that vary in fundamental frequency. Adults responded to calls that were farthest in frequency from the habituated stimulus as if they were completely different, whereas juveniles demonstrated comparatively low levels of discrimination for calls the same distance away. These results indicate that the ability to discriminate between long calls based on fundamental frequency improves with age. Thus as in human infants, the ability to perceive vocal communication sounds may depend on experience with vocal sounds and experience-dependent maturation of neural circuits [3], [10], [23].

For the most part 35-day birds were more similar to 20-day birds than adults but some differences between 20- and 35-day birds were observed. The behavioral tests showed that juvenile birds showed some improvement in the ability to perceptually distinguish vocal calls by 35 days of age: although the performance of 35-day birds did not improve significantly as a function of distance from the habituating stimulus (Fig. 3B, left), their scores were not different from either adults or 20-day birds at the third step, suggesting a perceptual ability intermediate between 20-day birds and adults. Interestingly, frequency resolution matures early in mammals, whereas other factors, such as frequency discrimination of two sequential tones and detection of amplitude modulation, mature relatively late [22], [52]. The ability of zebra finches to distinguish vocal calls based on fundamental frequency, as studied here, suggests a protracted period of maturation.

In addition, neural discrimination scores of 35-day birds, but not 20-day birds, showed a significant increase with distance from the habituating stimulus (Fig. 3B, right), indicating that NCM neurons develop better discriminative ability as birds acquire a memory of the tutor song. Neural discrimination of learned vocal calls is further improved in adult birds, suggesting that tuning of NCM neurons to fundamental frequencies and/or to other aspects of vocal calls is refined throughout the period of vocal learning. Thus, vocal practice and auditory-motor integration during later stages of vocal learning, as well as auditory experience, may contribute to the development of both perceptual skills and to neural tuning in NCM neurons.

The striking correspondence we observed between neural and behavioral measures of discrimination indicates that NCM neurons are becoming tuned in parallel with the refinement of perceptual distinctions between vocal calls of different fundamental frequencies. The similarity between the results of the neural and behavioral assays raises the possibility that refinements in neural processing within NCM may be causally related to behavioral improvements in the ability to perceptually distinguish vocal calls and is consistent with the idea that differences between age groups depended on neural processing rather than systematic differences in attention or other factors. The current results do not bear on the question of the possible contributions of experience-independent maturation of auditory circuits versus experience-dependent learning and neural refinement to the improvement of perceptual skills. Studies in mammals, including humans, have shown different time courses for perception of different auditory parameters [22], but we are not aware of any studies that have measured developmental changes in parameters such as frequency or amplitude tuning in songbird brain. In relation to the present results, a better understanding of the development of tonal receptive fields in songbird auditory circuits, as well as how developmental experience with both vocal and non-vocal sounds shapes auditory discrimination and neural coding, is needed to advance our understanding of increased perceptual acuity for vocal calls.

Interestingly, 20-day zebra finches, which are just leaving the nest for the first time, were able to discriminate vocal calls based on their fundamental frequency, even at frequencies closest to the habituated stimulus. The current results cannot resolve whether this initial ability to distinguish between calls is innate, based on auditory experience in the nest, or some combination. Previous studies have shown that fledgling male songbirds develop begging calls with individual-specific acoustic characteristics while still in the nest, which can be used by parents to recognize offspring [54]. In addition, begging calls are altered by deafening, influenced by lesions of vocal motor cortex, and incorporated into vocal babbling sounds as development progresses [55]. This pattern suggests that vocal learning in songbirds may be initiated well before birds begin to memorize tutor sounds. The overall pattern of results we observed is consistent with the idea that the perception of vocal communication signals depends on both innate predispositions and a phase of early learning. The present results are similar to those of human studies showing that infants begin life with predispositions based on innate auditory skills, and subsequently improve their ability to perceptually distinguish phonetic contrasts for sounds expressed in their native language as a first step in the process of vocal learning [3], [10], [15]. This perceptual learning is thought to be an important component of the ability to group similar sounds into functional categories, and to discriminate between elementary categories of sounds.

A basic precept of neural development is that experience can sculpt patterns of neural connectivity during sensitive periods to influence emerging behaviors [20], [56]. Kuhl has suggested a “neural commitment” hypothesis, in which early experience with vocal sounds produces patterns of neural connectivity that encode those vocal sounds and constrain subsequent vocal learning [4], [16], [17]. The strong correspondence observed here between behavioral and neural measures of discrimination of vocal calls during development is consistent with this hypothesis. For example, 35-day birds showed significant improvement in discriminative ability for vocal sounds farther removed from the habituated stimulus in neural tests, which correlated with their behavioral performance (which was intermediate between 20-day and adult birds in both cases). This pattern suggests that experience-dependent refinement of connectivity of NCM neurons during the first two weeks of vocal learning may underlie early improvements in behavioral perception. NCM receives strong projections from thalamo-recipient auditory cortex and is reciprocally interconnected with parts of auditory thalamus and also with higher-order auditory cortical regions that project directly to sensorimotor vocal control regions such as the High Vocal Center (HVC) [57], [58], [59]. In addition, ERK signaling in NCM is necessary for formation of a memory of tutor song during vocal learning [46]. Thus, it seems likely that NCM may be an important neural locus underlying perceptual learning for vocal sounds, an idea that must be tested by future studies.

One possible example of neural commitment in the songbird brain is provided by a striking refinement in the pattern of neural connectivity between cortical vocal-control nuclei which occurs soon after the onset of song learning: the projection from a cortico-basal ganglia pathway into vocal motor cortex has little topographic organization in 20-day birds, but achieves an adult-like topographic map by 35 days [24], [25] (Fig. 1). This substantial remodeling of axonal connectivity requires normal auditory experience during this phase, suggesting that hearing a range of vocal sounds is important for sculpting a pattern of neural connectivity that supports subsequent vocal learning. The salient re-modeling of this pathway suggests the possibility of an early stage of auditory-motor mapping when young animals are learning the correspondence between articulatory gestures and their acoustic consequences. An early phase of learning could entail changes in specificity within the neural substrate that are essential for subsequent phases of vocal acquisition, and that also impede learning new vocal patterns [3], [14]. If so, then improvements in perceptual ability in both humans and songbirds may depend, at least in part, on early forms of sensorimotor integration in which juveniles learn the sounds produced by specific motor gestures as neural connections are refined.

This idea is consistent with the fact that both human and songbird studies have shown tight links between perception and production [60], [61], [62], [63]. For example, microstimulation of motor cortex representing tongue versus lip in humans facilitates perception of phonemes associated with the corresponding articulator, and inhibits perception of contrasting phonemes [64]. In songbirds, single vocal-control neurons respond to the bird’s own song with the same precisely timed pattern of action potentials, regardless of whether birds are hearing their song played back or producing it [65]. Furthermore, songbirds have auditory-vocal mirror neurons, which respond the same way when birds are producing their own song, as well as when they are hearing playback of the same note sequence produced by another bird [66].

The trend observed here for neural activity in NCM to provide a more sensitive measure of developmental changes in perceptual ability than behavior is intriguing, in the sense that similar results are seen in human infants: event-related potentials appear to provide a more sensitive index of speech perception than do behavioral measures [27]. The apparent increased sensitivity of neural measures in NCM may be attributable to the fact that multiple brain regions, including regions afferent and efferent to NCM, may also contribute to perception of vocal sounds. If so, then other brain regions may develop on a different time scale than NCM. For example, selective auditory tuning in some cortical sensorimotor brain regions for vocal control does not mature until ∼60 days [67].

An interesting result of this study is that by the time birds have reached adulthood they show better discrimination of calls with FFs below 650 Hz. Female zebra finches produce long calls in this range (Fig. S1) and because female calls are simpler than male calls, FF is likely to be a major cue in individual female call recognition. Male zebra finches are able to recognize the call of their own mate and fine-scale frequency discrimination within this range may be helpful in discriminating between similar female calls.

## Materials and Methods

We developed both a behavioral and a neural assay to measure the ability of zebra finches to discriminate between synthetic long calls that varied in Fundamental Frequency (FF). Both of these assays relied on a habituation/dishabituation paradigm; during the first phase a stimulus with a specific FF was presented repeatedly until the response to that stimulus habituated. During the test phase that immediately followed habituation, a range of test stimuli varying in FF were presented (the habituated stimulus and all test stimuli were randomly interleaved). Three ages of male zebra finches were tested: 20 days (range = 19–22 days; birds of this age have just fledged from the nest and are at the onset of learning tutor sounds), 35 days (range = 34–36 days; at this age birds have memorized tutor sounds and are just beginning to produce babbling vocalizations) and adult (100-day birds are producing a stable vocal pattern of songs and calls). All birds were reared in our aviaries up until the day of testing and thus had normal social and song experience. All procedures were specifically approved by the University of Southern California Institutional Animal Care and Use Committee and followed the recommendations in the Guide for the Care and Use of Laboratory Animals of the National Institutes of Health.

### Call Stimuli

Stimuli used in this experiment were synthetic female long calls (Fig. S1) that were systematically varied in FF. D.S. Vicario kindly provided us with synthetically generated calls in the form of harmonic stack stimuli 310 ms in duration, with amplitude and harmonic emphasis derived from natural female calls [43]. We selected one of these synthetic calls (550 Hz FF), and stretched or compressed it such that the fundamental and all the harmonics were shifted to create new calls with different FFs (Adobe Audition). Three stimulus sets were created from the 550 Hz synthetic call; the synthetic call in the middle of each stimulus set was used as the habituating stimulus. The first stimulus set included synthetic calls of 360, 410, 440, 460, **480**, 500, 520, 550 and 600 Hz, the second set included 500, 550, 600, 625, **650**, 675, 700, 750 and 800 Hz synthetic calls and the third set included 620, 680, 740, 770, **800**, 830, 860, 920 and 980 Hz synthetic calls (bold indicates habituating stimulus).

### Behavioral Assay

Zebra finches that hear a natural or synthetic long call respond by emitting one or more long calls of their own [28], [42], [44]. This call-back response served as the dependent measure in our behavioral assay of perception. Individual birds were isolated in a small cage within a sound isolation box in a quiet room. The door of the box was left open and a speaker was placed ∼100 cm from the front of the bird’s cage; calls were played at 60 dB measured with a decibel meter from the front of the cage (SPL, A weighting). Playback of call stimuli was controlled using Spike 2 software and an analog/digital converter (Power 1401, Cambridge Electronic Design) and amplified with an audio amplifier (RadioShack). Call-back responses were recorded through a microphone (Audio Technica PRO-63), amplified 1500×, band-pass filtered (200–10,000 Hz; Brownlee Precision, model 440 amplifier), and digitized and stored using Spike 2.

Adult birds were removed directly from their home aviary and placed in the isolation box for 24–72 hours to allow them to acclimate to the testing environment. Individual 20-day and 35-day birds were removed from their nesting aviary and placed in the cage with their father for 4–8 hours; the father was removed from the cage within one hour before lights off and the juvenile was tested the next day between 10 AM and 2 PM. We conducted two separate behavioral experiments with different sets of birds; in the first experiment birds were habituated with a 480 Hz stimulus and tested with stimuli ranging from 410–550 Hz and in the second experiment birds were habituated to a 650 Hz stimulus and tested with stimuli ranging from 550–700 Hz (test stimuli came from the first two stimulus sets above). The habituating stimulus was presented every 10±2 seconds until the bird did not call back on 2 out of 3 presentations; as soon as this criterion was met, 5 interleaved repetitions of each test stimulus and the habituated stimulus were presented in random order (10±2 seconds). Calls that were higher in frequency than the habituated stimulus and calls that were lower in frequency than the habituated stimulus were presented in separate tests such that each test included four calls (3 test stimuli and 1 habituated stimulus). The high and low frequency ranges were tested separately to keep behavioral tests relatively short and thus ensure that birds continued to call back throughout the testing period. The order of tests with higher-frequency trials versus lower-frequency trials was counterbalanced across birds and there was a gap of at least one hour between the two tests. The number of call-back responses that occurred between 50 ms and 3 seconds following the start of each stimulus was counted for each bird.

Different birds were used for each of the tests shown in Figure 2 (i.e., 3 different age groups for 2 separate behavioral tests plus 3 separate neural tests, for a total of 15 groups); the final number of birds tested in each age group during each test ranged between 8 and 10. Birds that did not call back to 5 repetitions of the initial habituating stimulus (i.e., when that stimulus was first presented) were not tested. In the call-back test that used 650 Hz as the habituating stimulus, the percentage of birds that were excluded was 64% for adults (18/28), 68% for 35-day birds (19/28), and 48% for 20-day birds (11/23). In the call-back test that used 480 Hz as the habituating stimulus, the percentage of birds that were excluded was 27% for adults (4/15), 52% for 35-day birds (11/21), and 48% for 20-day birds (10/21). In all cases, birds were excluded either because they called back very little or not at all during the initial habituation phase (86% of the total number of birds that were excluded fell in this category), or because they failed to show reliable habituation (14%).

### Electrophysiological Assay

Birds were removed directly from their home aviary, anesthetized with 1.5% isoflurane (inhalation) and placed in a stereotaxic apparatus. A stainless steel post was glued to the rostral skull with dental cement (Lang Dental) and cyanoacrylate. Birds were then placed in individual cages and returned to the main aviary; 20- and 35-day old birds were placed in cages with their fathers. After a period of at least 2 hours awake birds were wrapped in a cloth jacket and placed in a plastic tube in the stereotaxic apparatus; the head was immobilized via the steel post. A craniotomy was made over NCM using stereotaxic coordinates: anterior 0.6–0.8 mm, lateral 0.4 mm, depth 0.8–1.5 mm. This area represents the most dorso-caudal region of NCM, in which habituation to complex auditory stimuli such as birdsongs and calls has been reported to be robust [45], [50], [68]. A carbon fiber microelectrode (Carbostar-1, Kation Scientific; impedance 0.5–1.0 MΩ) was used to record multi-cellular activity. The signal was amplified (10,000×), band-pass filtered (300–3,000 Hz; DAM 80 amplifier, World Precision Instruments) and recorded using Spike 2 software (20 kHz sampling rate). An unfamiliar conspecific song was used as a search stimulus to ensure that the electrode was within NCM (spikes of medium, heterogeneous amplitude with a brisk response throughout the song presentation). Most birds heard fewer than 5–10 presentations of this search stimulus. The habituating stimulus was then presented 30 times followed immediately by 5 repetitions each of eight test stimuli and the habituated stimulus randomly interleaved with each other; the inter-stimulus interval was 10±2 seconds. Response strength was measured by subtracting the response rate (spikes/sec) during a 1-sec period before stimulus onset from the response rate during each stimulus presentation. Only one track was made per bird since the dishabituating stimuli were no longer novel following one test. At the end of the experiment a small electrolytic lesion was made by applying 8–12 µA for 5–10 seconds to mark the electrode location. None of the birds tested in the electrophysiological assay were excluded from analysis. Following recording, birds were deeply anesthetized with Equithesin (0.04 ml/10 g) and perfused through the heart with 0.7% saline followed by 10% formaldehyde. Brains were post-fixed, frozen-sectioned in the coronal plane at a thickness of 50 µm, and Nissl stained to verify the location of the recording site.

### Data Analysis

We normalized both the behavioral and the electrophysiological responses by calculating a discrimination score for each frequency tested: the response to the test stimulus minus the response to the habituating stimulus during the test (R_T_ – R_HT_) was divided by the initial response to the habituating stimulus during the habituation phase minus the response to the habituating stimulus during the test (R_HI_–R_HT_). This measure results in a score of 0 if the test stimulus elicits the same response as the habituated stimulus and a score of 1 if the test stimulus elicits a completely dishabituated response (i.e., identical to the initial response to the habituating stimulus). For the behavioral experiments the initial response to the habituating stimulus was based on the total number of call-backs during presentations 2–6 of the habituating phase. The response to the first call during the habituation phase was not used because birds sometimes gave many more calls to the first presentation than to any subsequent stimulus presentation (we interpreted this as a possible startle response). The response to each stimulus during the test phase was based on the total number of call-back responses that occurred within 3 sec from onset for the 5 presentations of each call. For the neural experiments the mean response strength of the first 5 stimulus presentations was calculated for the presentation of each stimulus during the habituation and test phases and the discrimination score was calculated from these mean response strength values.

We generated psychometric functions by combining data across experiments and plotting discrimination scores versus cents difference from the habituated stimulus. Cents are defined as the log_2_ of the ratio of two tones, and are used to measure small acoustic intervals (one octave is defined as 1200 cents; thus an octave spans 12 semi-tones of 100 cents each). We normalized the cents difference from the habituated stimulus by 498 cents (the largest difference tested) and calculated the slope of normalized cents versus discrimination score for each age based on all data points across individual birds. (Trendlines plotted in Fig. 3A show mean data points with the y-intercept set to zero for clarity.) We then grouped discrimination scores based on how many frequency steps away they were from the habituated stimulus. The first step included discrimination scores for FFs 20–30 Hz (64–74 cents) away from the habituated stimulus, the second step 40–60 Hz (125–151 cents), the third step 70–120 Hz (236–281 cents) and the fourth step 120–180 Hz (351–498 cents).

ANOVAs were used to test the significance of linear regressions (Fig. 3A). Non-parametric statistics were used to evaluate all other data. The main effect of age was evaluated using Kruskal-Wallis tests and individual group-wise comparisons were evaluated using Mann-Whitney tests (Figs 3B and 4A). Friedman tests for repeated measures were used to determine whether frequency steps had an effect on discrimination scores at each age and one-sample signed rank Wilcoxon tests were run to determine whether discrimination scores were different from zero (Fig. 3B). Within the 500–800 Hz range Wilcoxon signed rank tests were used to assess significant within-subject differences to responses below versus above 650 Hz (Fig. 4B).

## Supporting Information

Figure S1 Characteristics of Long Calls in Zebra Finches. Top: Frequency versus time spectrograms showing examples of a natural (left) and a synthetic (right) female long call, each with a fundamental frequency (FF) of 550 Hz. Bottom: distribution of the fundamental frequency of long calls from 19 adult males and 28 adult females recorded from our breeding population of zebra finches. The FF of female long calls ranged from 486–698 Hz with a mean of 591±9. The majority (89%) of female calls fell between 500 and 650 Hz. Male long calls begin with a brief fast-frequency modulation preceding a constant-frequency harmonic stack [30], [37]. We measured the FF of the constant-frequency portion of male long calls, which was more variable in fundamental frequency and ranged from 571–1400 Hz with a mean of 855±54 Hz. Most male long calls (89%) were higher than 650 Hz.
(TIF)

Figure S2 NCM activity during the habituation phase in an adult bird. Top: raw traces of multi-unit activity in NCM (caudo-medial nidopallium, an area of higher-level auditory cortex) during the first presentation of a long call with a fundamental frequency of 650 Hz (left) and the last (30^th^) presentation of that stimulus (right). PSTHs show the multi-unit response to playback of the call over the first 10 trials (left) versus the last 10 trials (right). Response strength in NCM decreased by 40% over the course of habituation from 168 spikes/sec over the first five trials to 103 spikes/sec over the last 5 trials. Bottom: This plot shows the response to repeated presentations of the 650 Hz stimulus; the habituation curve had a slope of −2.53.
(TIF)

Figure S3 Call-back behavior of male birds varies as a function of age but not as a function of call frequency. Total number of long calls (mean+SEM) given by three different age groups of birds in response to 20 repetitions each of a natural male call, a natural female call, and synthetic calls with fundamental frequencies ranging from 450 to 950 Hz. Means for each age averaged across all synthetic call frequencies are given at right. These birds were tested as controls, and were not used for any tests of dishabituation. Adult birds gave significantly more call-back responses to the female call than to the male call (Wilcoxon Signed Ranks test, *p* = 0.01). Adults called back slightly more to synthetic long calls within the range of fundamental frequencies of female calls (450–650 Hz) than to calls with higher frequencies; however there was no significant effect of frequency on number of call-back responses (Friedman test for repeated measures, *p* = 0.35). Thirty-five day birds showed a slight trend toward calling back more to the female call than to the male call (Wilcoxon, *p* = 0.07) and there was no effect of frequency on the call-back response to synthetic calls (Friedman, *p* = 0.86). Twenty day birds produced the same number of long calls regardless of frequency with no difference between the response to male and female long calls (Wilcoxon, *p* = 0.75) or across frequencies (Friedman, *p* = 0.13). In summary, birds did not call-back preferentially to synthetic calls of specific fundamental frequencies at any age. Age did influence the number of call-back responses produced; 20-day birds produced more calls than adults while 35-day birds produced fewer. The effect of age on number of call-back responses (averaged across all frequencies) was significant (Kruskal Wallis, *p* = 0.02). Individual comparisons showed a significant difference between 20-day birds and adults (*p* = 0.05) and 20 and 35-day birds (*p* = 0.01) but not between 35-day birds and adults (p = 0.12).
(TIF)

Figure S4 Number of trials to reach behavioral criterion and number of call-back responses during the habituation phase showed some effects of age. Left panels: 20-day birds tended to require a larger number of stimulus presentations to reach criterion for the 480-Hz habituation stimulus while adult birds tended to require a larger number of 650-Hz call presentations to reach the habituation criterion; see Results. Right panels: these graphs plot the average number of calls produced in each age group on the first trial used for analysis (i.e., trial 2; see Methods) and then at each quartile point throughout the habituation training phase (i.e., the number of trials to criterion was normalized to 0–100% and the number of calls given in response to the call stimulus at each quartile point was averaged across birds at each age for each of the two behavioral experiments). These data show that the initial number of call-back responses given to the habituation stimulus in both the 480- and 650-Hz tests was similar between 20-day birds and adults, whereas 35-day birds tended to produce fewer calls at the onset of habituation training. Thereafter, 20-day birds maintained a higher level of call-back responding throughout most of the habituation phase whereas the call-back rate of adult birds quickly dropped to match the 35-day rate.
(TIF)

Figure S5 Initial response strength in NCM does not vary as a function of call frequency or age. A: Response strength (mean ± SEM) recorded in NCM (caudo-medial nidopallium) in response to the three synthetic calls used as habituating stimuli; these birds were tested for dishabituation immediately after this habituation phase. As shown in previous studies the neural response to a repeated call decreased with each iteration of the stimulus [45], [50]. At all three ages the initial response to the synthetic call stimulus (fundamental frequency of 480, 650 or 800 Hz) was the same. We calculated the mean response strength to the first 5 and last 5 calls at each age within each frequency range. There was no significant effect of age on mean response strength to either the first 5 or the last 5 calls within any of the three frequency ranges tested (Kruskal-Wallis, *p* always >0.05). This pattern demonstrates that the response to repetitions of the synthetic calls in NCM was similar across age groups. There was no difference in the strength of the multi-unit response in NCM between synthetic calls of different FFs: Kruskal Wallis tests on the mean response strength over the first five call iterations revealed no effect of frequency in any age group (*p* always >0.80). These results show that stronger responses to the test stimuli compared to the habituated stimuli were not due to innate preferences for specific stimuli. However, there were age differences in the percentage decrease between the first 5 and the last 5 iterations of each call (see B), reflecting the fact that juvenile birds showed a greater decrement in response strength across stimulus repetitions than did adults. Kruskal Wallis tests showed a main effect of age for the 480 Hz stimulus (*p* = 0.05) and the 650 Hz stimulus (*p* = 0.01), but not for the 800 Hz stimulus (*p* = 0.15). Individual comparisons (Mann Whitney) for the 480 Hz call revealed that 35-day birds showed a larger response decrement relative to both 20-day birds and adults (*p* = 0.05 and *p* = 0.03, respectively). Comparisons for the 650 Hz call showed a larger response decrement in adult birds relative to 20-day birds (*p* = 0.003) and 35-day birds (*p* = 0.05).
(TIF)
