# Supplementary figures and images for: Development of Auditory-Vocal Perceptual Skills in Songbirds
Source: PLoS One. 2012 Dec 20;7(12):e52365. doi: 10.1371/journal.pone.0052365 (PMC3527493; doi:10.1371/journal.pone.0052365)

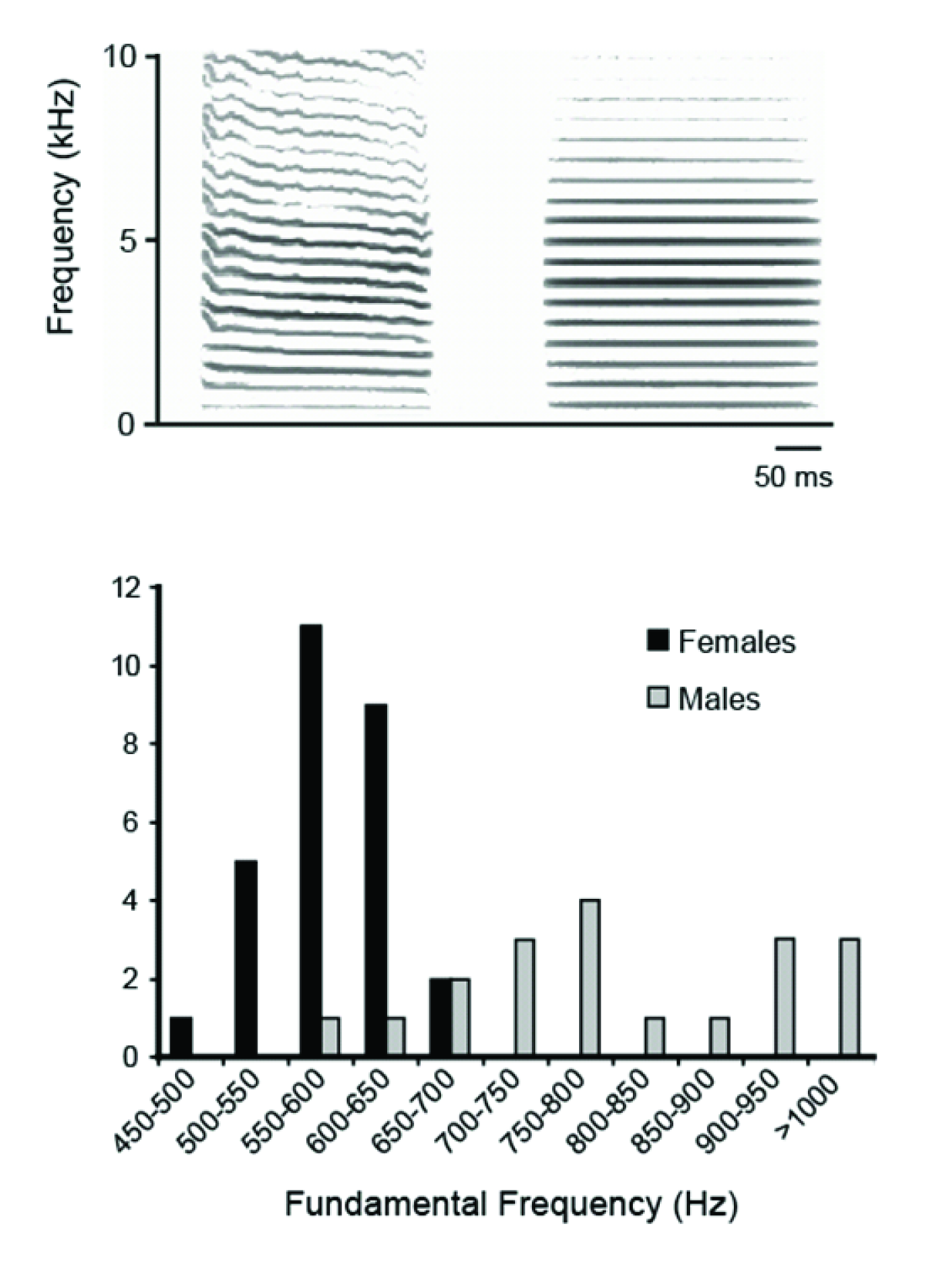

Supplement: Figure S1 — Characteristics of Long Calls in Zebra Finches. Top: Frequency versus time spectrograms showing examples of a natural (left) and a synthetic (right) female long call, each with a fundamental frequency (FF) of 550 Hz. Bottom: distribution of the fundamental frequency of long calls from 19 adult males and 28 adult females recorded from our breeding population of zebra finches. The FF of female long calls ranged from 486–698 Hz with a mean of 591±9. The majority (89%) of female calls fell between 500 and 650 Hz. Male long calls begin with a brief fast-frequency modulation preceding a constant-frequency harmonic stack [30], [37]. We measured the FF of the constant-frequency portion of male long calls, which was more variable in fundamental frequency and ranged from 571–1400 Hz with a mean of 855±54 Hz. Most male long calls (89%) were higher than 650 Hz. (TIF) [file pone.0052365.s001.tif]

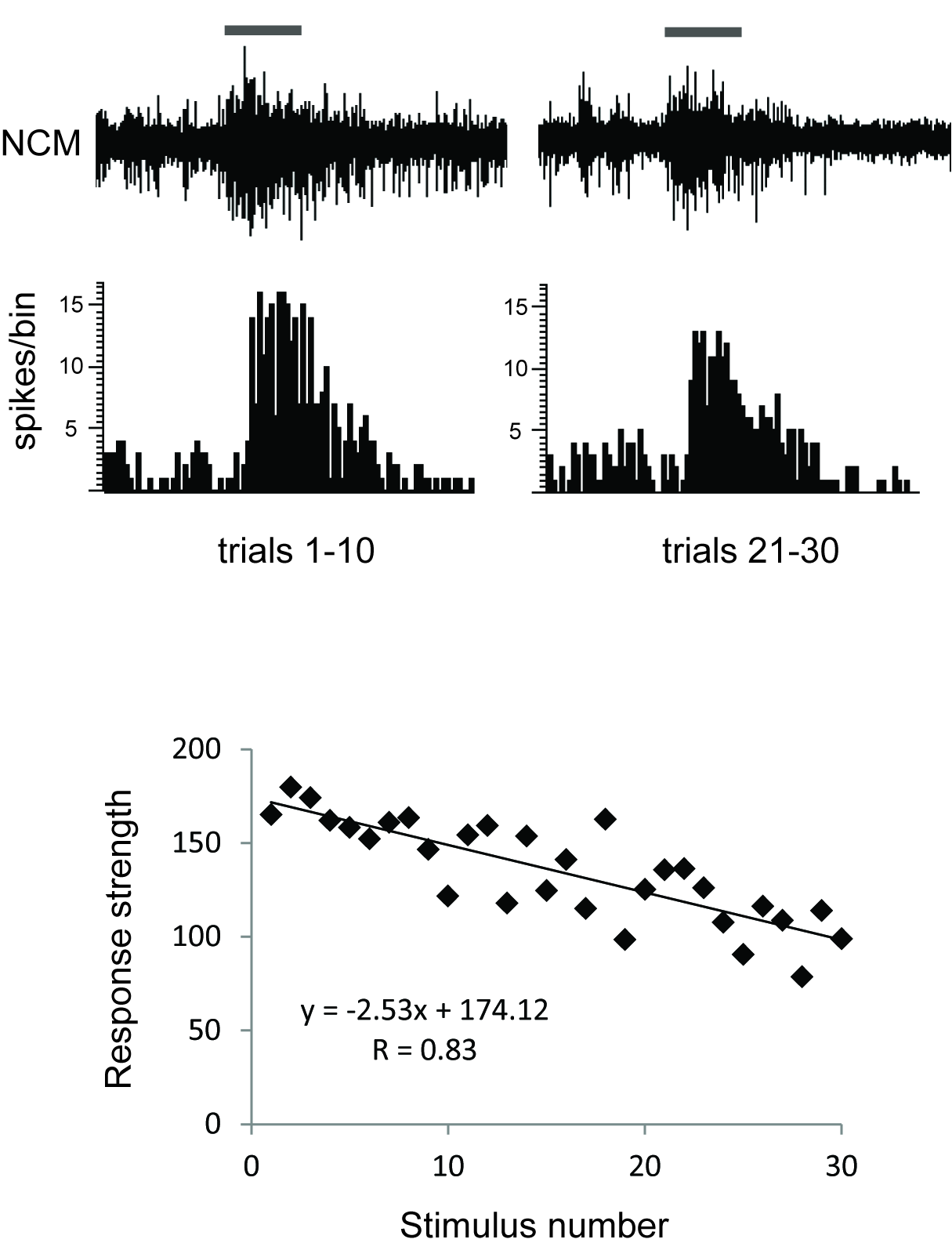

Supplement: Figure S2 — NCM activity during the habituation phase in an adult bird. Top: raw traces of multi-unit activity in NCM (caudo-medial nidopallium, an area of higher-level auditory cortex) during the first presentation of a long call with a fundamental frequency of 650 Hz (left) and the last (30th) presentation of that stimulus (right). PSTHs show the multi-unit response to playback of the call over the first 10 trials (left) versus the last 10 trials (right). Response strength in NCM decreased by 40% over the course of habituation from 168 spikes/sec over the first five trials to 103 spikes/sec over the last 5 trials. Bottom: This plot shows the response to repeated presentations of the 650 Hz stimulus; the habituation curve had a slope of −2.53. (TIF) [file pone.0052365.s002.tif]

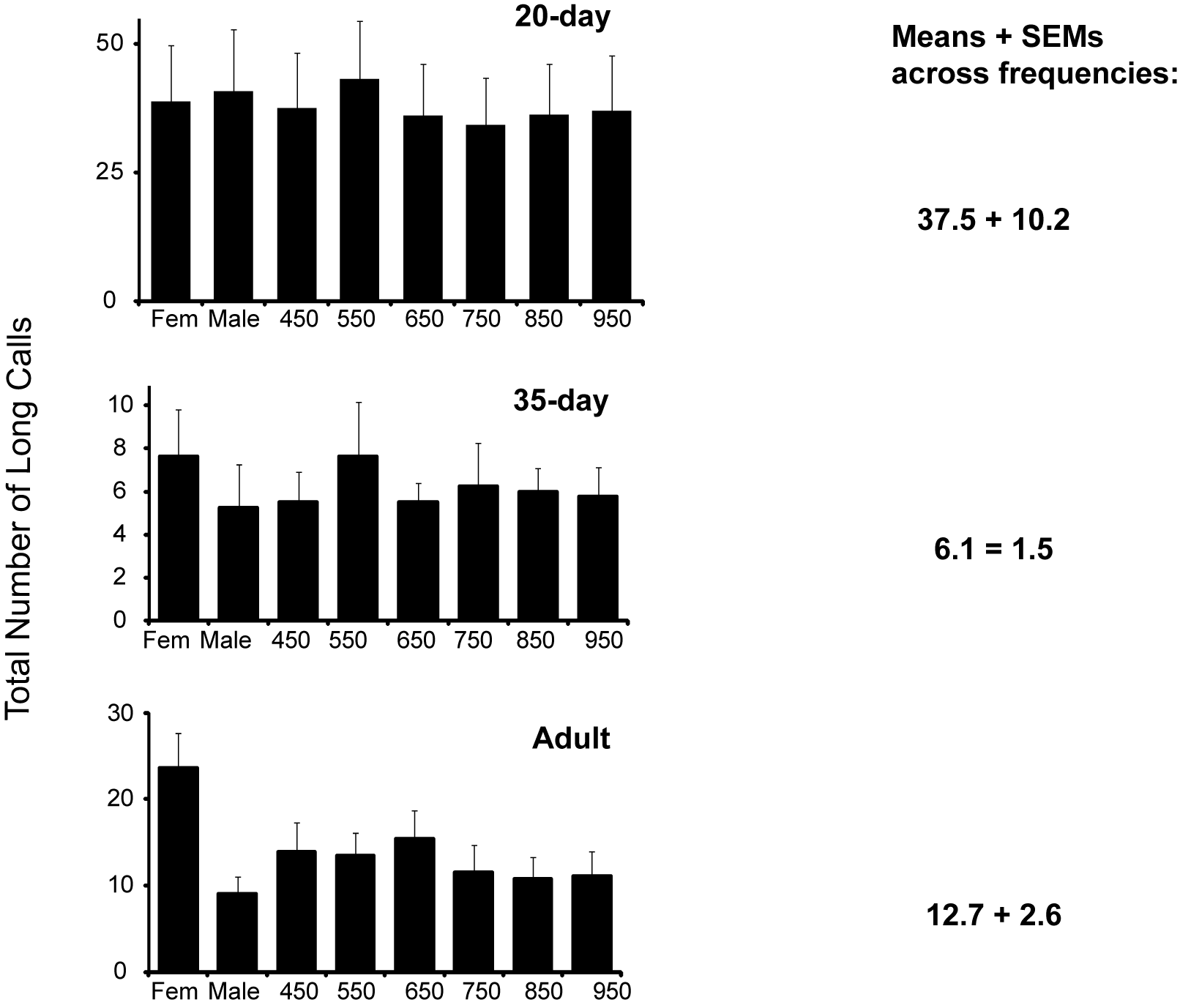

Supplement: Figure S3 — Call-back behavior of male birds varies as a function of age but not as a function of call frequency. Total number of long calls (mean+SEM) given by three different age groups of birds in response to 20 repetitions each of a natural male call, a natural female call, and synthetic calls with fundamental frequencies ranging from 450 to 950 Hz. Means for each age averaged across all synthetic call frequencies are given at right. These birds were tested as controls, and were not used for any tests of dishabituation. Adult birds gave significantly more call-back responses to the female call than to the male call (Wilcoxon Signed Ranks test, p = 0.01). Adults called back slightly more to synthetic long calls within the range of fundamental frequencies of female calls (450–650 Hz) than to calls with higher frequencies; however there was no significant effect of frequency on number of call-back responses (Friedman test for repeated measures, p = 0.35). Thirty-five day birds showed a slight trend toward calling back more to the female call than to the male call (Wilcoxon, p = 0.07) and there was no effect of frequency on the call-back response to synthetic calls (Friedman, p = 0.86). Twenty day birds produced the same number of long calls regardless of frequency with no difference between the response to male and female long calls (Wilcoxon, p = 0.75) or across frequencies (Friedman, p = 0.13). In summary, birds did not call-back preferentially to synthetic calls of specific fundamental frequencies at any age. Age did influence the number of call-back responses produced; 20-day birds produced more calls than adults while 35-day birds produced fewer. The effect of age on number of call-back responses (averaged across all frequencies) was significant (Kruskal Wallis, p = 0.02). Individual comparisons showed a significant difference between 20-day birds and adults (p = 0.05) and 20 and 35-day birds (p = 0.01) but not between 35-day birds and adults (p = 0.12). (TIF) [file pone.0052365.s003.tif]

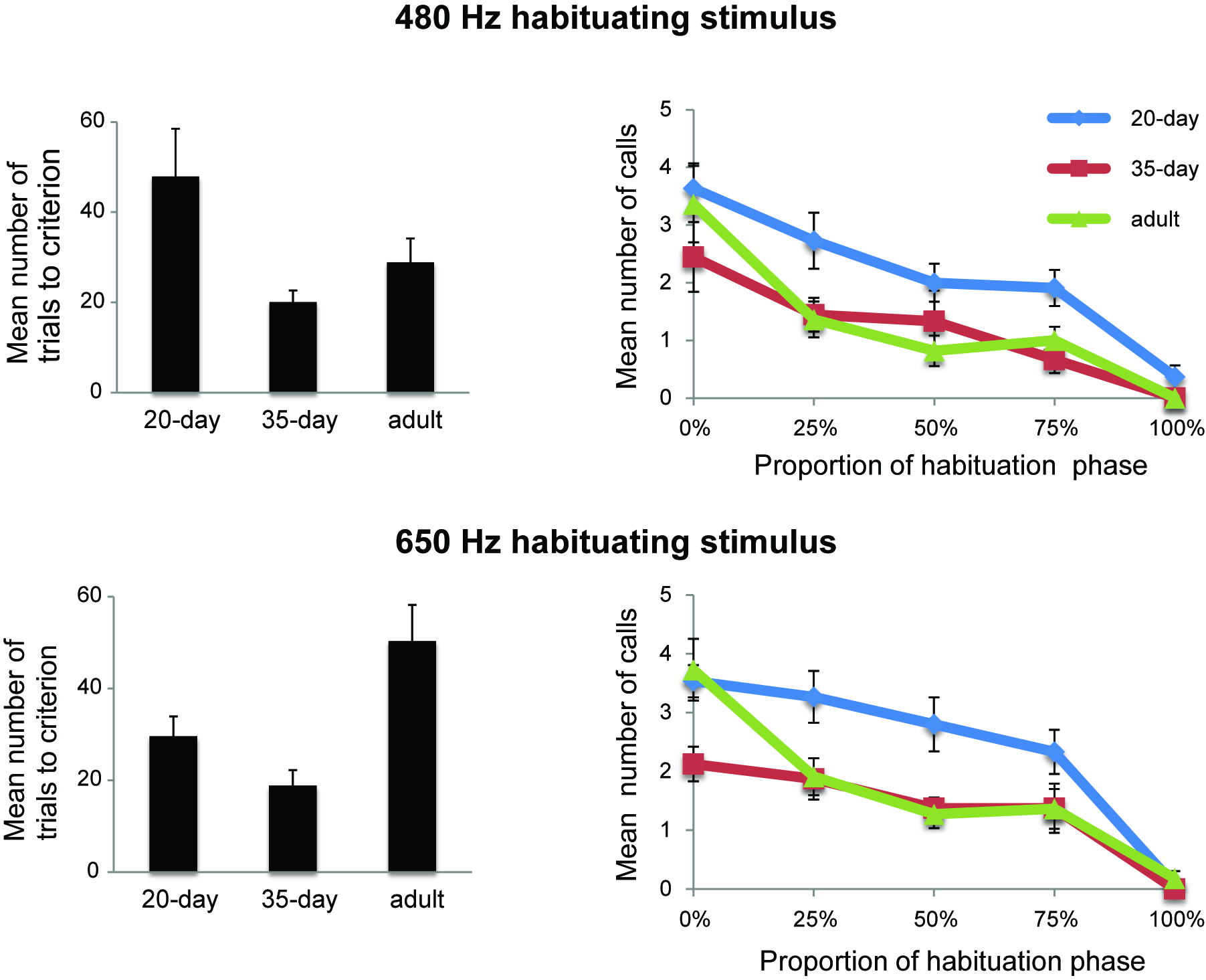

Supplement: Figure S4 — Number of trials to reach behavioral criterion and number of call-back responses during the habituation phase showed some effects of age. Left panels: 20-day birds tended to require a larger number of stimulus presentations to reach criterion for the 480-Hz habituation stimulus while adult birds tended to require a larger number of 650-Hz call presentations to reach the habituation criterion; see Results. Right panels: these graphs plot the average number of calls produced in each age group on the first trial used for analysis (i.e., trial 2; see Methods) and then at each quartile point throughout the habituation training phase (i.e., the number of trials to criterion was normalized to 0–100% and the number of calls given in response to the call stimulus at each quartile point was averaged across birds at each age for each of the two behavioral experiments). These data show that the initial number of call-back responses given to the habituation stimulus in both the 480- and 650-Hz tests was similar between 20-day birds and adults, whereas 35-day birds tended to produce fewer calls at the onset of habituation training. Thereafter, 20-day birds maintained a higher level of call-back responding throughout most of the habituation phase whereas the call-back rate of adult birds quickly dropped to match the 35-day rate. (TIF) [file pone.0052365.s004.tif]

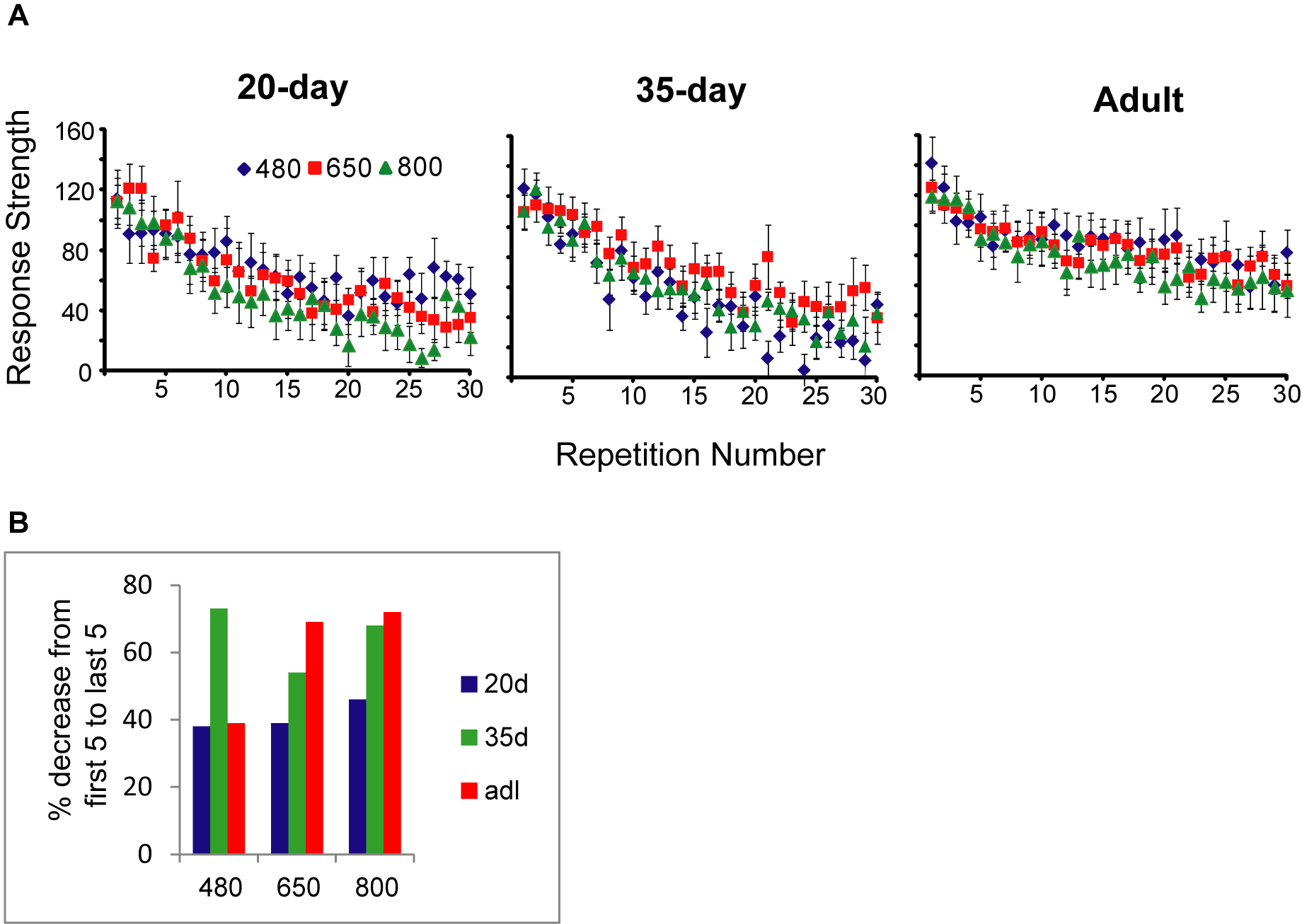

Supplement: Figure S5 — Initial response strength in NCM does not vary as a function of call frequency or age. A: Response strength (mean ± SEM) recorded in NCM (caudo-medial nidopallium) in response to the three synthetic calls used as habituating stimuli; these birds were tested for dishabituation immediately after this habituation phase. As shown in previous studies the neural response to a repeated call decreased with each iteration of the stimulus [45], [50]. At all three ages the initial response to the synthetic call stimulus (fundamental frequency of 480, 650 or 800 Hz) was the same. We calculated the mean response strength to the first 5 and last 5 calls at each age within each frequency range. There was no significant effect of age on mean response strength to either the first 5 or the last 5 calls within any of the three frequency ranges tested (Kruskal-Wallis, p always >0.05). This pattern demonstrates that the response to repetitions of the synthetic calls in NCM was similar across age groups. There was no difference in the strength of the multi-unit response in NCM between synthetic calls of different FFs: Kruskal Wallis tests on the mean response strength over the first five call iterations revealed no effect of frequency in any age group (p always >0.80). These results show that stronger responses to the test stimuli compared to the habituated stimuli were not due to innate preferences for specific stimuli. However, there were age differences in the percentage decrease between the first 5 and the last 5 iterations of each call (see B), reflecting the fact that juvenile birds showed a greater decrement in response strength across stimulus repetitions than did adults. Kruskal Wallis tests showed a main effect of age for the 480 Hz stimulus (p = 0.05) and the 650 Hz stimulus (p = 0.01), but not for the 800 Hz stimulus (p = 0.15). Individual comparisons (Mann Whitney) for the 480 Hz call revealed that 35-day birds showed a larger response decrement relative to both 20-day birds [file pone.0052365.s005.tif]
